# Segmentation of Leukoaraiosis on Noncontrast Head CT Using CT‐MRI Paired Data Without Human Annotation

**DOI:** 10.1002/brb3.70602

**Published:** 2025-06-10

**Authors:** Wi‐Sun Ryu, Jae W. Song, Jae‐Sung Lim, Ju Hyung Lee, Leonard Sunwoo, Dongmin Kim, Dong‐Eog Kim, Hee‐Joon Bae, Myungjae Lee, Beom Joon Kim

**Affiliations:** ^1^ Artificial Intelligence Research Center JLK Inc. Seoul Republic of Korea; ^2^ Department of Radiology University of Pennsylvania Philadelphia Pennsylvania USA; ^3^ Department of Neurology, Asan Medical Center University of Ulsan College of Medicine Seoul Republic of Korea; ^4^ Department of Radiology, Seoul National University College of Medicine Seoul National University Bundang Hospital Seongnam Republic of Korea; ^5^ Department of Neurology Dongguk University Ilsan Hospital Goyang Republic of Korea; ^6^ Department of Neurology, Seoul National University College of Medicine and Cerebrovascular Center Seoul National University Bundang Hospital Seongnam Republic of Korea

**Keywords:** computed tomography, deep learning, leukoaraiosis, magnetic resonance imaging, segmentation algorithm, white matter hyperintensities

## Abstract

**Objective:**

Evaluating leukoaraiosis (LA) on CT is challenging due to its low contrast and similarity to parenchymal gliosis. We developed and validated a deep learning algorithm for LA segmentation using CT‐MRIFLAIR paired data from a multicenter Korean registry and tested it in a US dataset.

**Methods:**

We constructed a large multicenter dataset of CT–FLAIR MRI pairs. Using validated software to segment white matter hyperintensity (WMH) on FLAIR, we generated pseudo‐ground‐truth LA labels on CT through deformable image registration. A 2D nnU‐Net architecture was trained solely on CT images and registered masks. Performance was evaluated using the Dice similarity coefficient (DSC), concordance correlation coefficient (CCC), and Pearson correlation across internal, external, and US validation cohorts. Clinical associations of predicted LA volume with age, risk factors, and poststroke outcomes were also analyzed.

**Results:**

The external test set yielded a DSC of 0.527, with high volume correlations against registered LA (*r* = 0.953) and WMH (*r* = 0.951). In the external testing and US datasets, predicted LA volumes correlated with Fazekas grade (*r* = 0.832–0.891) and the correlations were consistent across CT vendors and infarct volumes. In an independent clinical cohort (*n* = 867), LA volume was independently associated with age, vascular risk factors, and 3‐month functional outcomes.

**Interpretation:**

Our deep learning algorithm offers a reproducible method for LA segmentation on CT, bridging the gap between CT and MRI assessments in patients with ischemic stroke.

## Graphical abstract

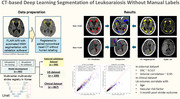

**Abbreviations**
CCC: concordance correlation coefficient
DSC: Dice similarity coefficient
FLAIR: fluid‐attenuated inversion recovery
HU: Hounsfield unit
LA: leukoaraiosis
mRS: modified Rankin Scale
NIHSS: National Institute of Health Stroke Scale
ReLU: rectified linear unit
WMH: white matter hyperintensity

## 1 Introduction

White matter hyperintensities (WMH), also referred to as leukoaraiosis (LA), are the most prevalent brain abnormalities identified on neuroimaging of elderly individuals (Black et al. 2009; Ryu et al. 2014). The presence and burden of LA are associated with an increased risk of stroke, dementia, depression, and poor outcomes following a stroke (Ryu et al. 2018, 2019, 2014, 2017). LA is most accurately detected using fluid‐attenuated inversion recovery (FLAIR) MRI. However, in clinical practice, LA is more frequently identified, and progression can be followed by CT rather than MRI due to the greater availability and accessibility of CT scanners.

Evaluating cerebral LA using CT is more challenging compared to MRI. The hypoattenuation characteristics of LA are less conspicuous against the background of white matter in the presence of gliosis or vasogenic edema on CT scans (Auriel et al. 2011). Although several LA scoring systems on head CTs are available (Scheltens et al. 1998), these systems typically permit only a limited number of ordinal ratings, rely on subjective visual criteria, and have poor associations with quantitative LA volume (Pantoni et al. 2002). Moreover, the interrater reliability of a visual rating scale using head CTs shows lower agreement (kappa of 0.5–0.6) compared with brain MRI (kappa around 0.8) (L. Chen et al. 2018; Pantoni et al. 2002; Wahlund et al. 2001). Therefore, while visual estimates of LA severity provide valuable prognostic information, they have limited sensitivity as diagnostic tools or markers of disease progression.

Recently, several studies have proposed deep learning approaches to automate LA segmentation on CT or MRI. van Voorst et al. (2024) developed a convolutional neural network trained on 245 CT scans with expert annotations, reporting a moderate Dice similarity coefficient (DSC) of 0.68, but the model's external performance dropped sharply to a DSC of 0.23, indicating poor generalizability across datasets. Pitkanen et al. (2020) used 147 CT–FLAIR MRI pairs to train a model, achieving high volume correlation (*r* = 0.94), though the evaluation was limited to the training set without external validation. L. Chen et al. (2018) applied an automated pipeline across CT and MRI modalities and found only modest agreement with expert‐segmented LA volumes (*r* = 0.71 for CT and *r* = 0.85 for MRI), highlighting the limitations of reproducibility in multicenter cohorts.

These approaches share common challenges: (1) reliance on manual annotations, which are inherently subjective and time‐consuming, especially for CT, where LA is difficult to delineate; (2) limited training sample sizes that restrict generalizability; and (3) poor spatial agreement in external datasets, undermining their clinical utility.

Recently, we developed software that automatically segments WMH on FLAIR MRI (H. Kim et al. 2024). Tested on an external validation dataset comprised of multicenter data (*n* = 6,031), the software showed a high DSC of 0.72 (H. Kim et al. 2024). In the current study, we aimed to develop a deep learning algorithm to automatically segment LA on noncontrast head CT scans. Using a CT‐MRI_FLAIR_ paired dataset, we implemented the validated software to segment WMH on FLAIR, then registered the segmentations on the noncontrast head CT, and subsequently trained the algorithm using CT scans without expert annotation. We externally validated this algorithm using an independent testing dataset and assessed its performance in a different ethnic cohort via a visual rating scale. Finally, in an independent clinical dataset, we examined the clinical implications of automatically predicted LA volumes in relation to risk factors and clinical outcomes following ischemic stroke.

Recent studies in brain imaging highlight complementary approaches to lesion characterization and clinical translation. Metabolomics‐based neuroimaging has revealed mechanisms of neurogenesis and axon regeneration after brain injury (Hu et al. 2023). Computational work has shown that strain rate estimation methods affect brain injury modeling outcomes (Zhan et al. 2025), while image‐guided hematoma evacuation underscores the importance of precise lesion localization (C. Zhang et al. 2021). These studies emphasize the value of multimodal imaging and robust validation—principles we apply in developing a reproducible, annotation‐free CT‐based LA segmentation method.

## 2 Materials and Methods

### 2.1 Datasets

**FIGURE 1:**
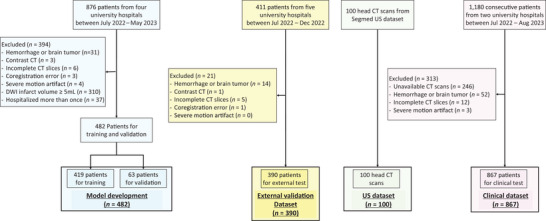
Study flow chart. DWI, diffusion‐weighted imaging.

This study originated from the Clinical Research Collaboration for Stroke in Korea (CRCS‐K), a nationwide web‐based registry that records patients with acute ischemic stroke or transient ischemic attack admitted to 20 stroke centers in South Korea (B. J. Kim et al. 2015; J. Kim et al. 2023; J. Y. Kim et al. 2019). From the imaging substudy, between July 2022 and May 2023, we included 876 patients with available CT‐MRI_FLAIR_ paired data for training and internal validation datasets from four university hospitals (Figure 1). We then excluded the following patients: duplicated due to recurrent stroke, large (> 5 mL) infarct core, severe motion artifact on CT or MRI, registration error, incomplete CT slices, contrast‐enhanced CT, and presence of hemorrhage or brain tumor. Infarct core volumes were measured on diffusion‐weighted imaging using verified in‐house software (Ryu, Kang, et al. 2023; Ryu et al. 2024). An infarct core volume threshold of > 5 mL was defined as an exclusion criterion to minimize differences between CT and FLAIR images, given the possibility of progression of ischemia and cytotoxic edema with LA on imaging.

For the external test dataset, 411 patients from five university hospitals were identified between July 2022 and December 2022; these cases did not overlap with those in the training dataset. The exclusion criteria were the same as for the training dataset, except duplicate cases due to recurrent stroke and patients with large infarct cores (> 5 mL) were included to evaluate the model's performance in a real‐world ischemic stroke dataset.

For the external US population dataset, we acquired 100 noncontrast head CTs along with their radiological impressions from Segmed, Inc. (Stanford, CA). All scans had protected health information, except for age and sex, removed from both the reports and DICOM tags. The cases included were drawn from both outpatient and emergency care settings. We filtered the scans based on the following criteria: (1) > 18 years old; (2) unenhanced; (3) without motion artifacts; (4) slice thickness ≥ 1.5 mm; (5) conducted using a standard convolutional kernel; (6) absence of intracranial hemorrhage or large transcortical infarcts; and (7) axial plane.

A clinical validation dataset was curated to evaluate the clinical relevance of automatically measured LA volumes on CT. From two comprehensive stroke centers in Korea (July 2022–August 2023), 1180 consecutive patients were identified. These institutions did not overlap with the centers from which the training and validation or external test datasets were collected. We excluded patients if: (1) a CT scan was not available, (2) presence of hemorrhagic transformation or brain tumor, (3) incomplete CT slices, and (4) severe motion artifact on CT. Using the initially acquired noncontrast head CT scans, we measured LA volumes using the algorithm. Demographic and clinical data were extracted from the prospective stroke registry. Modified Rankin Scale (mRS) scores at 3 months after stroke and admission, and National Institute of Health Stroke Scale (NIHSS) scores were collected as previously reported (Ryu, Chung, et al. 2023; Ryu et al. 2022, 2019, 2021). The study protocol was approved by the institutional review board of Seoul National University Bundang Hospital (B‐2307‐841‐303), and all subjects or their legal proxies provided written informed consent.

### 2.2 Imaging Protocols

For the training and internal validation dataset, the most frequent MRI vendor was Philips (*n* = 273, 56.6%; Table S1), followed by GE (*n* = 127, 26.4%) and Siemens (*n* = 82, 17.0%). The magnetic field strength was 3.0 Tesla (*n* = 415, 86.1%) and 1.5 Tesla (*n* = 67, 13.9%). Most patients had a slice thickness of 5 mm (*n* = 446, 92.5%). For noncontrast head CT scans, the most frequent CT vendor was Siemens (*n* = 262, 54.4%), followed by Philips (*n* = 208, 43.2%), and Canon (*n* = 11, 2.3%). Most patients had a slice thickness of 5 mm (*n* = 432, 89.6%) and underwent CT scans with a kVp of 120 (*n* = 443, 91.9%).

In the external testing dataset, the most frequent MRI vendor was Philips (*n* = 325, 83.3%), followed by Siemens (*n* = 39, 10.0%) and GE (*n* = 24, 6.2%). The magnetic field strength was 3.0 Tesla (*n* = 369, 94.6%) and 1.5 Tesla (*n* = 20, 5.1%). Most patients had a slice thickness of 5 mm (*n* = 287, 73.6%). For noncontrast head CT scans, the most frequent CT vendor was Siemens (*n* = 185, 47.4%), followed by GE (*n* = 91, 23.3%), Philips (*n* = 81, 20.8%), and Canon (*n* = 28, 7.2%). Most patients had a slice thickness of < 5 mm (*n* = 238, 61.0%) and underwent CT scans with a kVp of 120 (*n* = 282, 72.3%).

For the US dataset, the most frequent CT vendor was Siemens (*n* = 54, 54.0%), followed by GE (*n* = 40, 40.0%) and Canon (*n* = 5, 5.0%). Half of the patients had a slice thickness of 5 mm (*n* = 50, 50.0%), followed by 2.5 mm (*n* = 38, 38.0%). Additional details on imaging parameters are provided in Table S1.

### 2.3 Data Preprocessing and Preparation

The preprocessing procedure follows the default steps of the nnUNet framework. First, CT images are cropped to the smallest bounding box encompassing all nonzero regions. Next, the images are resampled using nearest neighbor interpolation to a target pixel spacing of *x* = 0.41 mm, *y* = 0.41 mm, and *z* = 5 mm, ensuring uniform voxel spacing across the dataset. This target spacing was determined by calculating the median pixel spacing in the training dataset. Finally, intensity normalization is applied based on the statistical properties of the entire dataset, given that CT scans use an absolute intensity scale. Intensity values are clipped to the 0.5th and 99.5th percentiles and *z*‐score normalized using the dataset's mean and standard deviation.

No human interactions were involved in the process of ground truth generation for CT images. We first generated WMH masks on FLAIR images utilizing validated software (H. Kim et al. 2024) and applied nonrigid registration using a statistical deformation model (Wouters et al. 2006) from FLAIR MRI to CT to transform the WMH mask. After registration, the LA mask on CT was thresholded at a probability of 0. Skull‐stripping was applied to both FLAIR and CT images beforehand for better alignment of image features.

### 2.4 Deep Learning Algorithms: nnUNet Framework

We employed the nnUNet framework (Isensee et al. 2021) and thus used the nnUNet architecture in a 2D configuration for deep learning. The 2D nnUNet adopts a similar architecture to the 2D UNet, consisting of an encoder–decoder structure with skip connections (Ronneberger et al. 2015). The encoder path comprises multiple convolutional blocks, each consisting of two 3 × 3 convolutional layers followed by a rectified linear unit (ReLU) activation and a 2 × 2 max‐pooling layer with a stride of 2. The decoder path mirrors the encoder path in a reverse manner. Each decoder block consists of an upsampling layer followed by two 3 × 3 convolutional layers and a ReLU activation. Between each encoder and decoder block, there is a skip connection with the same resolution in the decoder block. After the decoding step, a 1 × 1 convolutional layer is applied, followed by SoftMax activation to give the final output. For the learning and optimization step, we used the automated settings of the nnUNet framework, using the SGD optimizer with Nesterov momentum, an initial learning rate of 1e‐2, momentum of 0.99, and weight decay of 1e‐4 for 1000 epochs. Dice loss combined with binary cross entropy loss was used for the loss function. The final model consisted of 20 layers split between the encoder and decoder (Figure S1), and the final learning rate was 2e‐5. Data augmentation was applied during training, including random rotations, scaling, elastic deformation, intensity variations, and flipping. Early stopping was not applied, but we monitored the validation loss and Dice scores per epoch to assess overfitting.

### 2.5 Segmentation Performance Evaluation Metrics

The following metrics were used to evaluate the predicted LA segmentation against the registered LA mask on CT and the original, automated WMH segmentation on FLAIR:

$$\mathrm{Correlation}\mathrm{Coefficient}\;\left(r\right)=\frac{\sum \left(X_{i}-\bar{X}\right)\times \left(Y_{i}-\bar{Y}\right)}{\sqrt{\sum {\left(X_{i}-\bar{X}\right)}^{2}\;\;\times \sum {\left(Y_{i}-\bar{Y}\right)}^{2}}}.$$

$\;X_{i}=\;\mathrm{predicted}\;\mathrm{LA}\;\mathrm{volume}$.

$\;\bar{X}=\;\mathrm{mean}\;\mathrm{value}\;\mathrm{of}\;\mathrm{predicted}\;\mathrm{LA}\;\mathrm{volume}$.

$\;Y_{i}=\;\mathrm{Gound}\;\mathrm{truth}\;\mathrm{LA}\;\mathrm{volume}$.

$\;\bar{Y}=\;\mathrm{mean}\;\mathrm{value}\;\mathrm{of}\;\mathrm{Gound}\;\mathrm{truth}\;\mathrm{LA}\;\mathrm{volume}$.

$$\begin{array}{ccc} & & \mathrm{Concordance}\;\mathrm{correlation}\;\mathrm{coefficient}\;(\mathrm{CCC},\rho )\\ & & \;=\frac{2\times r\times \;\sigma_{X}\times \sigma_{Y}\;}{\sigma_{X}^{2}\times \sigma_{Y}^{2}+{\left(\mu_{X}+\mu_{Y}\right)}^{2}} \end{array}$$

*r* = correlation coefficient

σ= standard deviation, μ= mean

$$\mathrm{DSC}=\frac{2\mathrm{TP}}{2\mathrm{TP}+\mathrm{FP}+\mathrm{FN}},$$

where TP, FP, and FN indicate voxel‐level true positive, false positive, and false negative, respectively (Figure S2).

### 2.6 Experiment and Analysis

We implemented the network in Python 3.9.19 using PyTorch 2.3.1. The network (nnUNet) for training was trained on a GeForce RTX A6000 GPU with an 11.8 CUDA version, taking on average 60 s per epoch for 14,560/2205 slices (419/63 scans) with the training/validation split. We used a batch size of 12, automatically detected by the nnUNet framework.

After training with the training dataset, the coefficient of determination and concordance correlation coefficient were calculated to evaluate the LA segmentation performance against the external testing dataset. The 3D volume of a lesion is calculated by determining the volume of each voxel, based on pixel spacing and slice thickness. By summing the total number of voxels across all 2D slices and multiplying by the volume of each voxel, we obtain the total lesion volume. To evaluate the model's performance against expert manual annotation, an experienced vascular neurologist (W‐S. Ryu) with 20 years of experience manually segmented LA on CT scans with reference to FLAIR MRI images in 40 randomly selected cases from the external testing dataset. In the external test and the US dataset, an expert (W‐S. Ryu) visually rated the extent of LA on CT using a 4‐point Fazekas scale (Fazekas et al. 1987) (none, mild, moderate, and severe), blinded to predicted LA volumes.

### 2.7 Statistical Analysis

Data were presented as the mean ± SD or frequency (percentage or interquartile range [IQR]) as appropriate. Baseline characteristics between training and validation datasets versus the external test dataset were compared using *t*‐tests, rank‐sum tests, or chi‐square tests as appropriate. To compare the volumes of predicted LA with registered volumes of LA on CT and WMH volumes on FLAIR images, we utilized the Pearson correlation coefficient (*r*) and concordance correlation coefficient (CCC: *ρ*) with their 95% confidence intervals (CIs) (Lawrence and Lin 1989). To test the relationship between Fazekas' grade and predicted LA volumes, we used the Pearson correlation coefficient (*r*). The 95% CI for the performance metrics was calculated by determining the standard error of the mean for each metric. In the clinical study, associations between demographic and clinical variables and predicted LA volumes were tested using multiple linear regression analyses. The relationship between predicted LA volumes and 3‐month mRS score was assessed using multivariable ordinal logistic regression analysis. Because the proportional odds assumption was violated, we combined the mRS scores 5 and 6 into a single category in the analysis. *p* < 0.05 was considered statistically significant.

## 3 Results

### 3.1 Study Population

After exclusion, 482 CT‐MRI_FLAIR_ paired data from four university hospitals were used for training and internal validation (Figure 1). For the external testing dataset, 390 additional CT‐MRI_FLAIR_ paired data from the four stroke centers were included. The mean patient ages for the training/internal validation and external validation datasets were 68.1 (SD, 12.7) and 69.2 (SD, 13.5) years, and 33.2% and 47.4% were female, respectively (Table 1). The median of CT‐MRI exam intervals was 3.30 (IQR, 1.04–8.18) and 2.43 (IQR, 1.02–7.04) hours for the internal and external validation datasets, respectively. Median WMH volumes (IQR) on FLAIR MRI were 9.18 mL (4.62–18.5 mL) and 10.23 mL (4.56–22.60 mL), respectively.

**TABLE 1:** Baseline characteristics of training and validation dataset and external test dataset.

	Training and internal validation (*n* = 482)	External test (*n* = 390)	*p* value
Age, years (SD)	68.1 (12.7)	69.4 (13.5)	0.252
Sex, female (%)	160 (33.2%)	185 (47.4%)	< 0.001
Time interval between CT and FLAIR, hours (IQR)	3.30 (1.04–8.18)	2.43 (1.02–7.04)	0.022
Previous stroke (%)	88 (21.4%)	82 (19.9%)	0.565
WMH volume on FLAIR, mL (IQR)	9.18 (4.62–18.5)	10.23 (4.56–22.6)	0.091
Infarct volume on DWI, mL (IQR)	0.86 (0.16–2.37)	1.68 (0.39–8.25)	< 0.001
Slice thickness of CT (%)			< 0.001
< 5 mm	49 (10.1%)	238 (61.0%)	
5 mm	432 (89.6%)	137 (35.1%)	
> 5 mm	1 (0.21%)	14 (3.59%)	
CT Vendors^a^ (%)			< 0.001
GE	0 (0.0%)	91 (23.3%)	
SIEMENS	262 (54.4%)	185 (47.4%)	
Philips	208 (43.2%)	81 (20.8%)	
Canon (Toshiba)	11 (2.3%)	28 (7.2%)	
MRI Vendors^b^ (%)			< 0.001
GE	127 (26.4%)	24 (6.2%)	
SIEMENS	82 (17.0%)	39 (10.0%)	
Philips	273 (56.6%)	325 (83.3%)	
Canon (Toshiba)	0 (0.0%)	1 (0.3%)	

Abbreviations: FLAIR, fluid‐attenuated inversion recovery; IQR, interquartile range.

^a^ Data were missing in a patient in the training dataset and in five patients in the external test dataset.

^b^ Data were missing in a patient in the external test dataset.

### 3.2 Segmentation Performance of Deep Learning Algorithm

In the internal validation dataset (*n* = 63), the model achieved a DSC of 0.531 (95% CI, 0.497–0.564) versus registered LA on CT scans (Table S2). Volumetric analysis showed that the predicted LA volume on CT correlated with registered LA volume on CT (*r* = 0.957 and *ρ* = 0.898) and WMH volume on FLAIR (*r* = 0.951 and *ρ* = 0.813).

**FIGURE 2:**
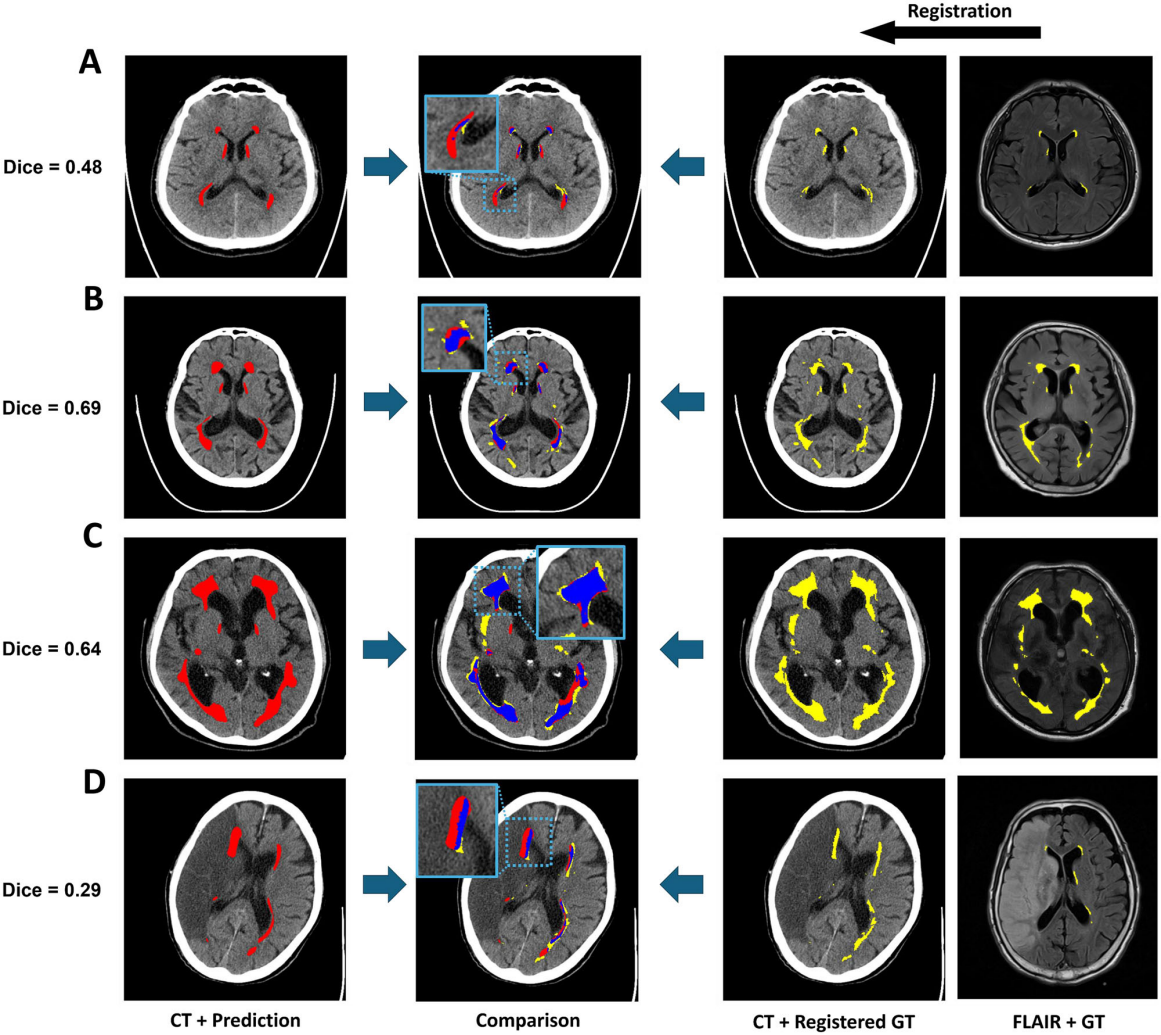
Representative cases of leukoaraiosis segmentation results using CT‐MRI paired data without human annotation. The first column shows CT images with predicted labels (red). The second column illustrates segmentation results from the deep learning model trained on CT‐MRI paired data, overlaid on CT images, with error maps highlighting discrepancies between predictions and pseudo‐ground truth (red: false positives; blue: overlap; yellow: false negatives). Insets provide zoomed‐in views of representative regions of disagreement. The third column shows CT images with pseudo‐ground truth labels (yellow), transferred from co‐registered FLAIR MRI, while the fourth column displays the corresponding FLAIR images with true lesion distributions. The pipeline demonstrates high visual concordance with MRI‐derived labels across varying degrees of lesion burden and anatomical locations.

**FIGURE 3:**
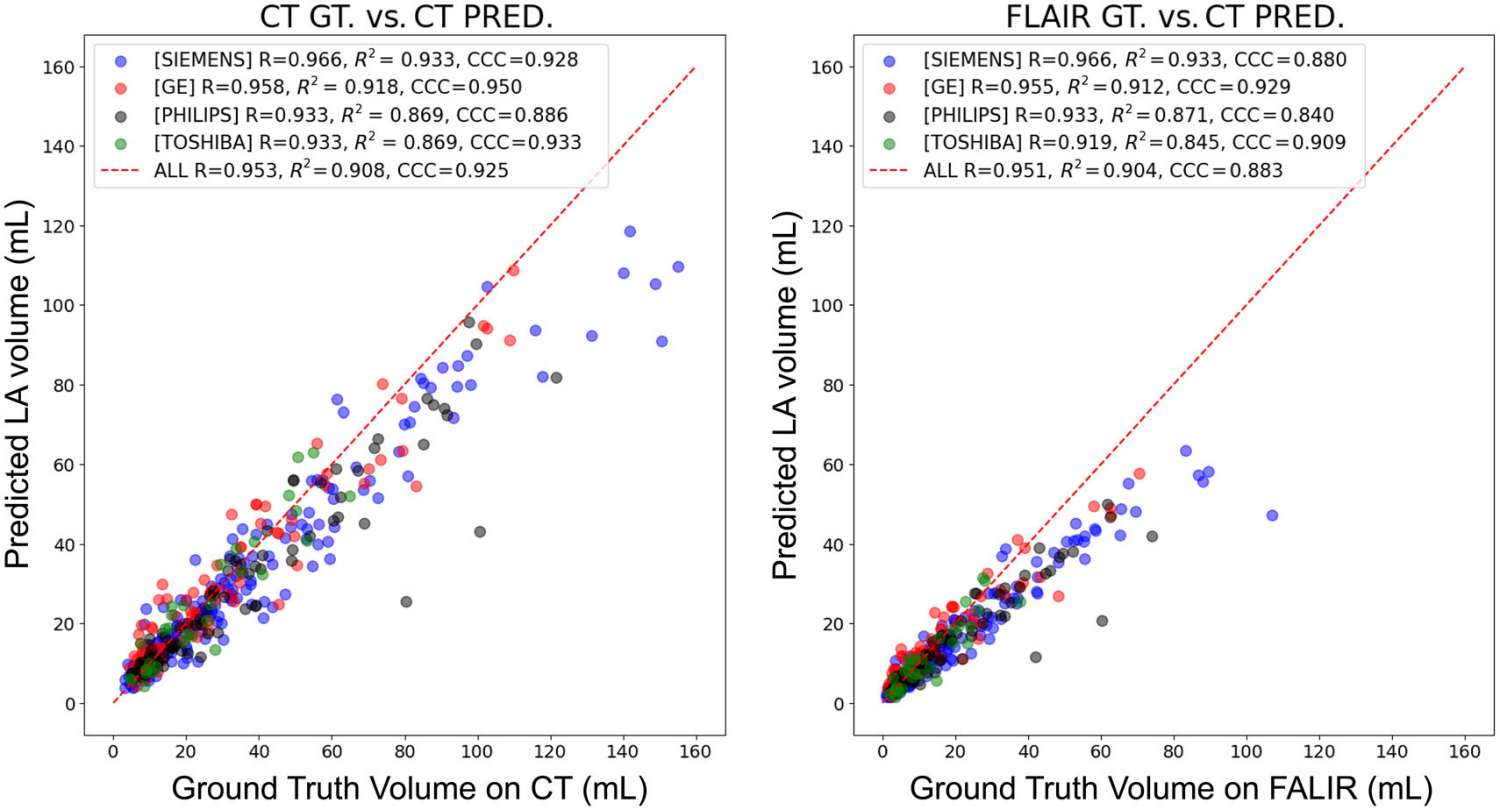
Volumetric correlation between automatically segmented leukoaraiosis volume on CT and ground truth on CT and MRI in the external test dataset. Dot plots showed a relationship between predicted versus registered leukoaraiosis volume (A) and between predicted leukoaraiosis volume on CT and predicted white matter hyperintensity volume on FLAIR (B). Each color represents a CT vendor. FLAIR, fluid‐attenuated inversion recovery; GT, ground truth.

**FIGURE 4:**
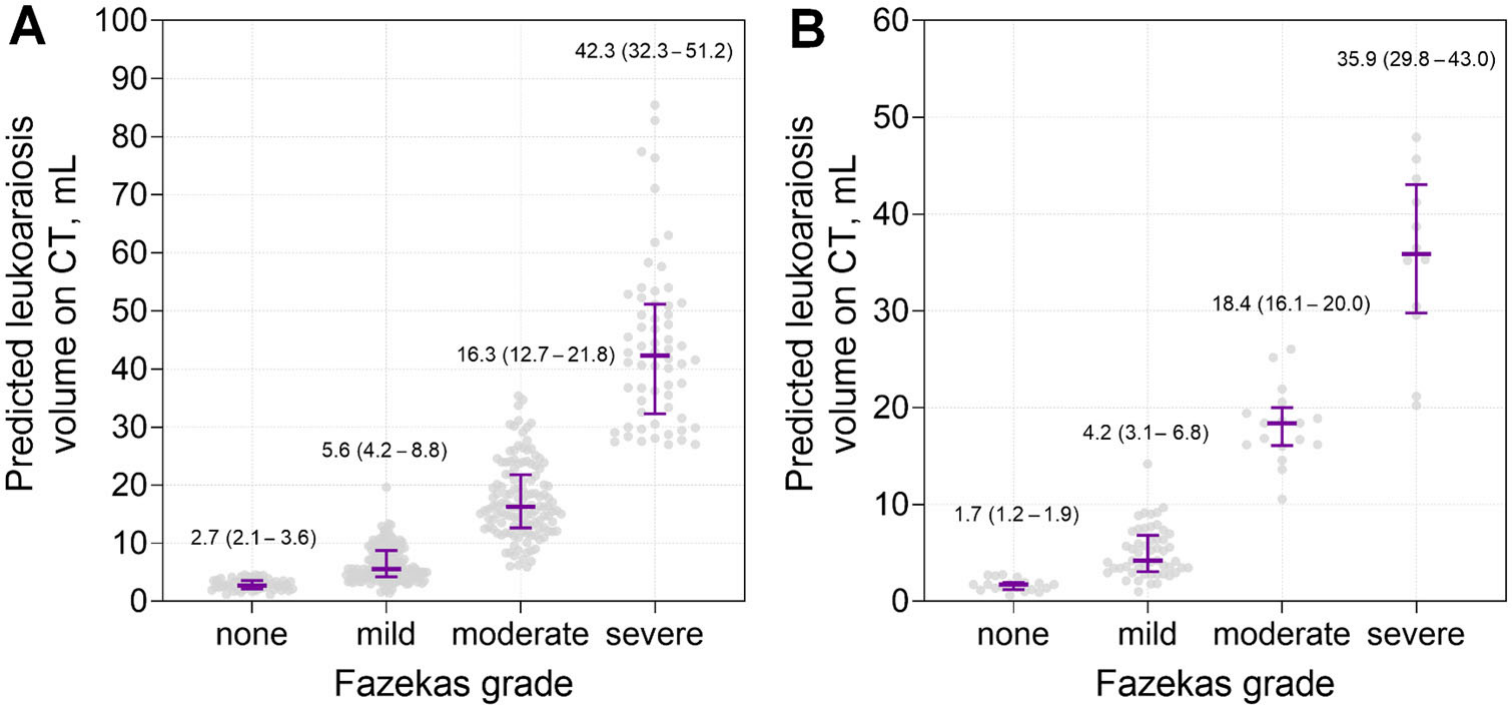
Volumetric correlation between the automatically segmented leukoaraiosis volume on CT and Fazekas grade in the external test dataset (A) and in the US population dataset (B). The numbers in the graph and purple lines (bars) indicate the median (interquartile range) of leukoaraiosis volumes for each Fazekas grade.

In the external test dataset, the DSC between predicted LA and registered LA on CT was 0.556 (0.545–0.566; Table 2). Representative cases with high DSC and low DSC between predicted LA and registered LA on CT in the external test dataset are shown in Figure 2. Volumetric analysis demonstrated excellent agreement between predicted LA volume and registered LA volume on CT (*r* = 0.953 and *ρ* = 0.925; Figure 3A) and good agreement between predicted LA volume and WMH volume on FLAIR images (*r* = 0.951 and *ρ* = 0.883; Figure 3B). With the increase of LA or WMH volumes, DSC increased in both internal validation and external test datasets (Figure S3). In 40 randomly selected cases with manual segmentation, the predicted LA volumes again demonstrated good agreement with manual segmentation (*ρ* = 0.858; Figure S4). In addition, predicted LA volumes in the external testing dataset were strongly correlated with Fazekas grade (*r* = 0.832; *p *< 0.001; Figure 4A).

**TABLE 2:** Performance of deep learning algorithms segmenting leukoaraiosis on brain CT.

		All	SIEMENS	GE	PHILIPS	TOSHIBA
		390	185 (47.4%)	91 (22.1%)	81 (20.8%)	28 (7.17%)
CT prediction vs. CT GT	*r* ^2^	0.908 (0.890 – 0.926)	0.933 (0.920 – 0.946)	0.918 (0.886 – 0.949)	0.869 (0.845 – 0.894)	0.869 (0.845 – 0.894)
	*r*	0.953 (0.943 – 0.961)	0.965 (0.954 – 0.974)	0.958 (0.937 – 0.972)	0.932 (0.897 – 0.956)	0.932 (0.865 – 0.966)
	CCC	0.925 (0.906 – 0.942)	0.928 (0.914 – 0.941)	0.950 (0.938 – 0.962)	0.886 (0.819 – 0.952)	0.933 (0.902 – 0.963)
	DSC	0.556 (0.545 – 0.566)	0.564 (0.548 – 0.580)	0.545 (0.524 – 0.566)	0.558 (0.536 – 0.579)	0.534 (0.493 – 0.574)
CT prediction vs. FLAIR GT	*r* ^2^	0.904 (0.868 – 0.909)	0.933 (0.920 – 0.946)	0.912 (0.869 – 0.942)	0.871 (0.846 – 0.895)	0.845 (0.816 – 0.873)
	*r*	0.951 (0.941 – 0.960)	0.966 (0.955 – 0.975)	0.955 (0.933 – 0.970)	0.933 (0.898 – 0.957)	0.919 (0.840 – 0.960)
	CCC	0.883 (0.856 – 0.908)	0.880 (0.837 – 0.924)	0.929 (0.907 – 0.951)	0.840 (0.754 – 0.926)	0.909 (0.830 – 0.988)

*Note*: Data were presented as estimates (95% confidence interval).

Abbreviations: CCC, concordance correlation coefficient; DSC, Dice similarity coefficient; FLAIR, fluid‐attenuated inversion recovery; GT, ground truth.

In the US population (mean age 64.6 ± 15.2 years [range: 24–90 years], 58.0% male), the predicted LA volumes showed a strong correlation with Fazekas grade (*r* = 0.891; *p* < 0.001; see Figure 4B).

### 3.3 Subgroup Analysis After Stratification by CT Vendors and Infarct Core Volume on DWI

The model exhibited consistent segmentation performance independent of CT vendors (Table 2) with excellent agreement (*r* = 0.932–0.965 and *ρ* = 0.886–0.950). In comparison, the predicted LA volume was correlated with WMH on FLAIR with *ρ* ranging from 0.840 to 0.929 (Figure S5).

In the external validation dataset, after stratification by infarct core volume (> 10 mL [*n* = 99; median 0.82 mL; IQR 0.24–2.78 mL] versus ≤ 10 mL [*n* = 312; median 37.0 mL; IQR 16.7–63.5 mL]), the model showed excellent agreement with registered LA volume on CT in both groups (*r* = 0.959 and *r* = 0.946, respectively; Figure S6). In comparison to WMH volumes on FLAIR, the model exhibited good agreement (*r* = 0.937 and *r* = 0.939, respectively) in both groups.

### 3.4 Clinical Study Using Automatically Measured LA Volumes on CT

After exclusion (Figure 1), 867 consecutive patients with ischemic stroke were included in the clinical study. The mean age was 69.3 years (SD, 13.0), and 39.2% were female (Table S3). The median predicted LA volume was 11.2 mL (IQR, 6.2–20.5 mL). Age was strongly associated with predicted LA volumes (coefficient 0.436, *p* < 0.001; Figure S7). Multiple linear regression analysis showed that age, prior stroke, and atrial fibrillation were independently related to LA volumes (Table S4). Hypertension was independently associated with LA volumes in younger patients (< 70 years) but not in elderly patients (≥ 70 years). In ordinal logistic regression analysis, the third, fourth, and fifth quintiles of LA volumes were incrementally associated with higher 3‐month mRS scores, respectively (Table 3). After adjusting for covariates, the association between LA quintiles and mRS scores was slightly attenuated but remained significant, with adjusted odds ratios of 1.59 (95% CI, 1.08–2.36) and 1.65 (95% CI, 1.10–2.46) for the fourth and fifth quintiles, respectively.

**TABLE 3:** Univariate and multivariable ordinal logistic regression analysis between quintiles of leukoaraiosis volumes and modified Rankin Scale score at 3 months.

	Range of leukoaraiosis volume, mL	Crude odds ratio (95% CI)	*p* value	Adjusted^a^ odds ratio (95% CI)	*p* value
1st quintile (*n* = 173)	0–5.77	Reference		Reference	
2nd quintile (*n* = 173)	5.78–9.26	1.35 (0.92 – 1.96)	0.12	1.16 (0.78 – 1.71)	0.46
3rd quintile (*n* = 173)	9.27–14.63	1.59 (1.09 – 2.31)	0.015	1.49 (1.01 – 2.19)	0.045
4th quintile (*n* = 173)	14.65–24.16	1.77 (1.21 – 2.58)	0.003	1.59 (1.08 – 2.36)	0.02
5th quintile (*n* = 174)	24.17–90.17	2.14 (1.46 – 3.12)	< 0.001	1.64 (1.10 – 2.46)	0.016

*Note*: Nagelkerke *R* ^2^ = 0.127 (for the multivariable model).

^a^ Adjusted for age, admission National Institutes of Health Stroke Scale score, sex, body mass index, hypertension, diabetes, hyperlipidemia, smoking, atrial fibrillation, coronary artery disease, and revascularization therapy.

## 4 Discussion

In the present study, a deep learning algorithm that automatically segments LA on head CT exams was developed using a CT‐MRI_FLAIR_ paired dataset without human annotation and externally validated in an independent, multicenter, multi‐vendor dataset. Moreover, we assessed the algorithm's efficacy in the US dataset using the visual rating scale. The predicted LA volumes on CT exhibited excellent agreement with WMH volumes on MRI across multiple CT vendors, showing generalizability. The predicted LA segmentations correlated well with manual segmentations outlined by an expert and a visual rating scale in both external testing and US datasets. Using a third clinical dataset, we show that the predicted LA volumes are indeed associated with vascular risk factors and stroke outcome.

Several studies have reported on deep learning algorithms for segmenting LA on CT scans (L. Chen et al. 2018; Pitkanen et al. 2020; van Voorst et al. 2024). L. Chen et al. (2018) demonstrated that the automated LA volume correlation at MRI was 0.85 and at CT imaging was 0.71 when compared with LA volumes segmented by experts, which is lower compared to our results. Pitkanen et al. (2020) developed a convolutional neural network algorithm using 147 paired CT‐MRIFLAIR images and reported a volumetric correlation of 0.94. However, they validated the algorithm using the same training data. van Voorst et al. (2024) developed an algorithm using 245 CT exams with expert annotations and reported a DSC of 0.68. However, the performance of the algorithm performed poorly in external validation testing, with a DSC of 0.23. Our algorithm, trained on a large CT‐MRI_FLAIR_ paired dataset, exhibited robust performance on an external dataset with DSC ranging from 0.54 to 0.60 and represents high performance for an externally verified LA segmentation algorithm.

Our results showed that the visual grading system correlates with quantitative LA volume, suggesting it can be a valuable tool for assessing LA burden. However, we also observed an overlap in LA volumes across grades in both Korean and US populations. Visual scoring systems for LA on CT, despite being widely used, are limited by their reliance on subjective visual criteria and the resultant variability in interrater reliability (Pantoni et al. 2002; Wardlaw et al. 2013). This variability can hinder accurate diagnosis and monitoring of disease progression. In contrast, the deep learning algorithm developed in this study offers objectivity and reproducibility. By eliminating human subjectivity, the algorithm enhances diagnostic accuracy and provides a reliable tool for assessing LA. This advancement is particularly important for large‐scale studies and clinical trials where reproducibility in LA measurement is crucial. Furthermore, quantitative LA assessments could support individualized care by enabling clinicians to monitor patients over time.

A key finding of the deep learning algorithm's validation involved testing its performance across multiple CT vendors. The algorithm demonstrated high CCC values, ranging from 0.905 to 0.953, indicating excellent cross‐vendor agreement. Consistent performance across different imaging vendors ensures that the algorithm can be widely adopted and provide reliable LA segmentations. This eliminates the need for image harmonization within and across institutions. This universality is a significant step towards standardizing LA assessment in clinical practice.

In the external testing dataset, the predicted LA volumes on CT significantly correlated with WMH volumes on FLAIR MRI. This correlation is crucial as it validates the algorithm's effectiveness in translating the more precise measurements typically obtained from MRI into the more commonly available CT scans (Abdalkader et al. 2023). In addition, CT scans remain the primary modality for patients presenting with neurological symptoms, although MRI is superior to CT in the diagnosis of stroke (Mullins et al. 2002). The ability to accurately assess LA on CT, using an algorithm that correlates well with MRI‐derived volumes, bridges the gap between the two imaging modalities. A strong correlation between predicted LA volumes and Fazekas grade in both Korean and US populations further supported the generalizability of our algorithm. Furthermore, using an independent clinical dataset, we demonstrated associations between automatically measured LA volume on CT and both risk factors and clinical outcomes after ischemic stroke, consistent with the known literature in studies using FLAIR MRI (Ryu et al. 2014, 2017). These results bolster the reliability and reproducibility of our algorithm, enhancing patient management where MRI is not readily available (Cabral Frade et al. 2022).

Our CT‐based algorithm tends to underestimate WMH volumes seen on FLAIR MRI due to CT's lower sensitivity for detecting LA (Wardlaw et al. 2015). This discrepancy occurs because subtle high‐signal intensities visible on FLAIR MRI may not be detected on CT. Despite this limitation, manual annotation of LA on CT is challenging and labor‐intensive due to its subtle appearance, underscoring the value of automated methods. More importantly, head CT scans are significantly more accessible than MRI globally (Aderinto et al. 2023). In clinical settings with limited MRI availability, our algorithm offers a practical means of quantifying LA burden, a measure correlated with adverse stroke outcomes, cognitive decline, and other conditions.

In the present study, we developed an algorithm for segmenting LA on CT without human annotation. By utilizing CT‐MRI_FLAIR_ paired data, the algorithm eliminates the need for labor‐intensive manual annotations, thereby streamlining the segmentation process. This approach ensures a consistent and objective analysis, free from the variability and potential biases inherent in human annotations in outlining obscure LA on CT (Y. Chen and Joo 2021; Geva et al. 2019; Sylolypavan et al. 2023). The ability to accurately segment LA on CT without human intervention enhances the algorithm's efficiency and reliability, making it a valuable tool for clinical practice and large‐scale studies.

Beyond segmentation performance, recent studies emphasize diverse approaches to stroke research that complement imaging‐based biomarkers. For instance, neuroendoscopic techniques like the NESICH approach highlight the clinical value of anatomically guided hematoma evacuation (Wang et al. 2024). On the molecular side, curcumin‐primed mesenchymal stem cells and mangiferin have shown neuroprotective effects by modulating inflammation and lipid metabolism in ischemic models (Lan et al. 2024; H. Zhang et al. 2024). Systemic indicators such as the blood urea nitrogen‐to‐albumin ratio also predict stroke outcomes (Liu et al. 2024), while rare stroke mimics like adult‐onset neuronal ceroid lipofuscinosis underscore diagnostic challenges (Huang et al. 2024). These examples reflect the multidimensional nature of stroke care—ranging from surgical innovation to molecular and diagnostic refinement—within which our AI‐driven, annotation‐free LA segmentation tool offers scalable support for small vessel disease profiling.

A notable source of variability in studies using MRI‐derived labels is the heterogeneity introduced by different MRI vendors, field strengths, and imaging protocols. Such differences can affect signal intensity and spatial resolution, potentially introducing bias into lesion segmentation (Sylolypavan et al. 2023; Wardlaw et al. 2015). To address this, we employed a standardized preprocessing pipeline that included skull‐stripping and nonrigid registration using a statistical deformation model to align FLAIR MRI scans with their corresponding CT images (Wouters et al. 2006). This approach effectively harmonized anatomical features across modalities and vendors, reducing inter‐scan variability before label transfer. Importantly, the algorithm was trained solely on CT images with MRI‐derived masks, making it inherently vendor‐agnostic during inference. The consistent segmentation performance observed across CT subgroups—despite substantial variability in MRI acquisition—suggests that our cross‐modality registration strategy successfully mitigated vendor‐related bias. Similar registration‐based harmonization strategies have been shown to improve generalizability in other neuroimaging studies (Hoving et al. 2018; Wouters et al. 2006), and our findings support their applicability to LA segmentation on CT.

While our model architecture (2D nnU‐Net) is widely used (Isensee et al. 2021), the novelty of this study lies in its annotation‐free training strategy. By transferring validated WMH masks from FLAIR MRI onto CT via deformable registration (Wouters et al. 2006), we trained the model without manual CT labels—overcoming challenges of subjectivity and poor visibility of LA on CT. Unlike prior studies that relied on expert annotations (L. Chen et al. 2018; van Voorst et al. 2024), our approach is scalable, reproducible, and achieved strong agreement with manual segmentation (*ρ* = 0.858). This label‐efficient method may offer a practical framework for CT‐based neuroimaging tasks where manual annotation is limited (Sylolypavan et al. 2023).

### 4.1 Limitations

Although the algorithm was validated using multicenter, multi‐vendor data, the training data was limited to Asian patients with ischemic stroke. However, in the US population, we observed a strong correlation between predicted LA volumes and Fazekas grade, indicating that our algorithm may be effective across different racial groups. Additionally, the exclusion criteria applied to the initial patient cohort, particularly the exclusion of patients with more than 5 mL of ischemic stroke on DWI, may have introduced bias into the training dataset. Nonetheless, subgroup analysis showed that the algorithm maintained its performance in patients with large infarcts on DWI, albeit with slightly lower accuracy compared to those with smaller infarcts. However, relatively low DSC in the external test limits the generalizability of our algorithm, requiring further validation in a large sample dataset. Even though our algorithm demonstrated a strong volume correlation between predicted LA volumes on CT and WMH volumes on FLAIR, regional similarity, as assessed by DSC, was relatively low. Weak DSC can be explained by the limitations of co‐registering different imaging modalities (Hoving et al. 2018), which limits the usability of our algorithm in studies where spatial correlation is crucial.

## 5 Conclusions

By providing a more accurate, reproducible, and accessible method for assessing LA, the proposed algorithm has the potential to improve patient care and outcomes. Its robustness across different imaging systems and validated correlation with MRI‐derived volumes enhance its clinical utility, making it a valuable tool for both routine clinical practice and research.

## Author Contributions

**Wi‐Sun Ryu**: conceptualization, investigation, writing – original draft, writing – review and editing, data curation, formal analysis, validation. **Jae W. Song**: writing – original draft, writing – review and editing, visualization. **Jae‐Sung Lim**: writing – original draft, writing – review and editing, data curation, formal analysis. **Ju Hyung Lee**: methodology, software, writing – review and editing, investigation, validation. **Leonard Sunwoo**: writing – review and editing, data curation, formal analysis, validation, visualization. **Dongmin Kim**: project administration, resources, writing – review and editing. **Dong‐Eog Kim**: writing – review and editing, data curation, investigation, resources, supervision. **Hee‐Joon Bae**: data curation, supervision, writing – review and editing, project administration. **Myungjae Lee**: project administration, writing – review and editing, writing – original draft, supervision, data curation, software, formal analysis, validation, resources. **Beom Joon Kim**: writing – review and editing, writing – original draft, supervision, data curation, project administration, visualization.

## Conflicts of Interest

Wi‐Sun Ryu, Ju Hyung Lee, Dongmin Kim, and Myungjae Lee are employees of JLK Inc., Republic of Korea. The other authors declare no conflicts of interest.

## Peer Review

The peer review history for this article is available at https://publons.com/publon/10.1002/brb3.70602

## Supporting information



Supporting Information

## Data Availability

Data supporting the findings of this study are available on request from the corresponding author. The data are not publicly available due to privacy or ethical restrictions.
