# Supplementary material for: Segmentation of Leukoaraiosis on Noncontrast Head CT Using CT‐MRI Paired Data Without Human Annotation
Source: Brain Behav. 2025 Jun 10;15(6):e70602. doi: 10.1002/brb3.70602 (PMC12152255; doi:10.1002/brb3.70602)
Supplement: Supplementary file 1 — Supporting Information [file BRB3-15-e70602-s001.docx]

Supplemental Material

**Segmentation of leukoaraiosis on noncontrast head CTs using CT-MRI paired data without human annotation**

Ryu et al.

Table S1. Imaging parameters in training, external test and US datasets

|  |  | Training and internal validation (n = 482) | External dataset  (n = 390) | US dataset  (n=100) |
| --- | --- | --- | --- | --- |
| CT | Vendors |  |  |  |
|  | Siemens | 262 (54.4 %) | 185 (47.4%) | 54 (54.0%) |
|  | GE | 0 | 91 (23.3%) | 40 (40.0%) |
|  | Philips | 208 (43.2 %) | 81 (20.8 %) | 1 (1.0%) |
|  | Canon | 11 (2.3 %) | 28 (7.2 %) | 5 (5.0%) |
|  | Thickness, mm |  |  |  |
|  | < 5mm | 49 (10.1 %) | 238 (61.0 %) | 50 (50.0%) |
|  | 5mm | 432 (89.6 %) | 137 (35.1 %) | 50 (50.0%) |
|  | > 5mm | 1 (0.21 %) | 14 (3.59 %) | 0 |
|  | Pixel spacing | 0.336 ~ 0.613 | 0.355 ~ 0.662 | 0.332 ~ 0.742 |
|  | Matrix |  |  |  |
|  | 320 ~ <512 | 0 | 75 (19.2%) | 0 |
|  | 512 | 482 (100.0%) | 257 (65.9%) | 97 (97.0%) |
|  | 512< ~ 560 | 0 | 58 (14.9%) | 3 (3.0%) |
|  | kVp |  |  |  |
|  | 90 | 1 (0.2%) | 0 | 0 |
|  | 100 | 37 (7.7%) | 15 (3.9%) | 3 (3.0%) |
|  | 110 | 0 | 1 (0.3%) | 5 (5.0%) |
|  | 120 | 443 (91.9%) | 282 (72.3%) | 49 (49.0%) |
|  | 130 | 1 (0.2%) | 3 (0.8%) | 12 (12.0%) |
|  | 140 | 0 | 89 (22.8%) | 31 (31.0%) |
| FLAIR MRI | Vendors |  |  |  |
|  | Siemens | 82 (17.0 %) | 39 (10.0 %) |  |
|  | GE | 127 (26.4 %) | 24 (6.2%) |  |
|  | Philips | 273 (56.6 %) | 325 (83.3 %) |  |
|  | Thickness, mm |  |  |  |
|  | 3 | 2 (0.4%) | 10 (2.6%) |  |
|  | 4 | 34 (7.1%) | 92 (23.6%) |  |
|  | 5 | 446 (92.5%) | 288 (73.8%) |  |
|  | Spacing between slices, mm |  |  |  |
|  | ≤5 | 36 (7.5%) | 94 (24.1%) |  |
|  | <5 ~ 6 | 220 (45.6%) | 283 (72.6%) |  |
|  | <6 ~ 7.5 | 226 (46.9%) | 14 (3.6%) |  |
|  | Pixel spacing | 0.234 ~ 0.500 | 0.199 ~ 0.898 |  |
|  | Matrix |  |  |  |
|  | 256~500 | 0 | 42 (10.8%) |  |
|  | 500~600 | 426 (88.4%) | 338 (86.7%) |  |
|  | 600~1024 | 56 (11.6%) | 10 (2.6%) |  |
|  | Magnetic strength |  |  |  |
|  | 1.5 | 67 (13.9%) | 20 (5.1%) |  |
|  | 3 | 415 (86.1%) | 369 (94.9%) |  |
|  | Repetition time, ms | 4800 ~ 11000 | 4800 ~ 12000 |  |
|  | Echo time, ms | 82 ~ 147 | 76 ~ 396 |  |

FLAIR=Fluid-attenuated inversion recovery.

Table S2. Performance of deep learning algorithms segmenting leukoaraiosis on brain CT in the internal validation dataset (n = 63)

|  | | Estimates (95% confidence interval) |
| --- | --- | --- |
| CT prediction vs. CT GT | r^2^ | 0.916 (0.878 - 0.954) |
|  | r | 0.957 (0.930 – 0.974) |
|  | CCC | 0.898 (0.845 – 0.950) |
|  | DSC | 0.531 (0.497 - 0.564) |
| CT prediction vs. FLAIR GT | r^2^ | 0.906 (0.863 – 0.948) |
|  | r | 0.951 (0.921 – 0.971) |
|  | CCC | 0.813 (0.748 – 0.878) |

GT=ground truth; CCC=Concordance correlation coefficient; DSC=Dice similarity coefficient; FLAIR=fluid-attenuated inversion recovery.

Table S3. Baseline characteristics of population for clinical study

| Age, mean ± SD | 69.3 ± 13.0 |
| --- | --- |
| Male | 527 (60.8%) |
| Onset to NCCT, hours, median (IQR) | 6.8 (2.1 – 23.8) |
| Initial NIHSS score, median (IQR) | 2 (0 – 4) |
| Body mass index, kg/m^2^, mean ± SD | 24.1 ± 3.6 |
| Pre-stroke mRS < 2 | 853 (98.4%) |
| Stroke subtype |  |
| Large artery atherosclerosis | 234 (27.0%) |
| Small vessel occlusion | 74 (8.5%) |
| Cardioembolism | 191 (22.0%) |
| Undetermined | 252 (29.1%) |
| Other determined | 19 (2.2%) |
| Transient ischemic attack | 97 (11.2%) |
| Prior history of stroke | 167 (19.3%) |
| Coronary artery disease | 76 (8.8%) |
| Hypertension | 520 (60.0%) |
| Diabetes | 274 (31.6%) |
| Hyperlipidemia | 251 (29.0%) |
| Atrial fibrillation | 193 (22.3%) |
| Current smoking | 181 (20.9%) |
| Revascularization therapy | 136 (15.7%) |
| 3-month mRS |  |
| 0 | 280 (32.3%) |
| 1 | 187 (21.6%) |
| 2 | 163 (18.8%) |
| 3 | 94 (10.8%) |
| 4 | 65 (7.5%) |
| 5 | 45 (5.2%) |
| 6 | 33 (3.8%) |
| Predicted LA volume, mL, median (IQR) | 11.2 (6.2 – 20.5) |

Data are presented as number (percentage). NCCT=noncontrast CT; IQR=interquartile range; mRS=modified Rankin Scale; LA=leukoaraiosis.

Table S4. Multivariable relationship between risk factors and automatically predicted leukoaraiosis volume on noncontrast CT after stratification by the median of age (≤69 years and ≥70 years)

|  | All (n = 846) | | < 70 (n =380) | | ≥70 (n = 466) | |
| --- | --- | --- | --- | --- | --- | --- |
|  | Standardized  Coefficient | *P* | Standardized  Coefficient | *P* | Standardized  Coefficient | *P* |
| Age | 0.485 | < 0.001 | 0.399 | < 0.001 | 0.383 | 0.001 |
| Sex | 0.638 | 0.48 | 1.231 | 0.24 | 0.178 | 0.90 |
| Prior stroke | 5.905 | < 0.001 | 3.986 | 0.001 | 7.693 | < 0.001 |
| Coronary artery disease | 0.366 | 0.80 | -2.118 | 0.25 | 1.845 | 0.36 |
| Hypertension | 0.561 | 0.53 | 2.109 | 0.023 | -1.041 | 0.48 |
| Diabetes | -0.004 | 0.99 | 0.292 | 0.77 | -0.508 | 0.72 |
| Hyperlipidemia | -0.260 | 0.78 | -1.497 | 0.12 | 0.903 | 0.56 |
| Smoking | 0.089 | 0.94 | 0.785 | 0.43 | -0.866 | 0.72 |
| Atrial fibrillation | -2.351 | 0.019 | -0.578 | 0.65 | -3.267 | 0.022 |


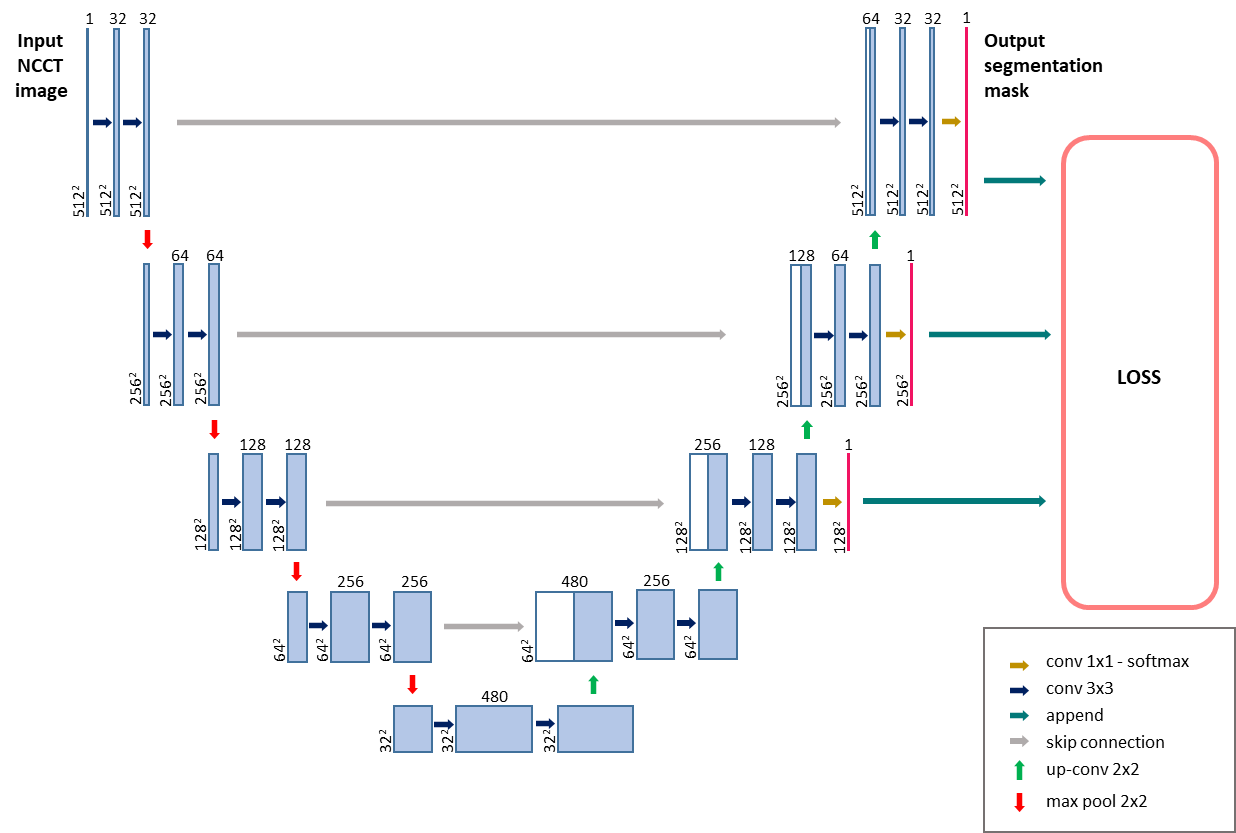


Figure S1. Schematic representation of nnUNet 2D model architecture.

Encoder 1: 32 filters, 3x3 kernel, Encoder 2: 64 filters, 3x3 kernel, Encoder 3: 128 filters, 3x3 kernel, Encoder 4: 256 filters, 3x3 kernel**,** Encoder 5: 512 filters, 3x3 kernel, Decoder 5: 512 filters, 3x3 kernel (with up-convolution, 2x2 kernel), Decoder 4: 256 filters, 3x3 kernel (with up-convolution,2x2 kernel), Decoder 3: 128 filters, 3x3 kernel (with up-convolution, 2x2 kernel)**,** Decoder 2: 64 filters, 3x3 kernel (with up-convolution, 2x2 kernel), Decoder 1: 32 filters, 3x3 kernel, Output Layer: 1 filter, 1x1 kernel

Final learning rate: 2e-5.

Optimizer: SGD with Nesterov momentum (momentum = 0.99, weight decay = 3e-5)

Loss function: Dice Loss + Cross-Entropy Loss.

**
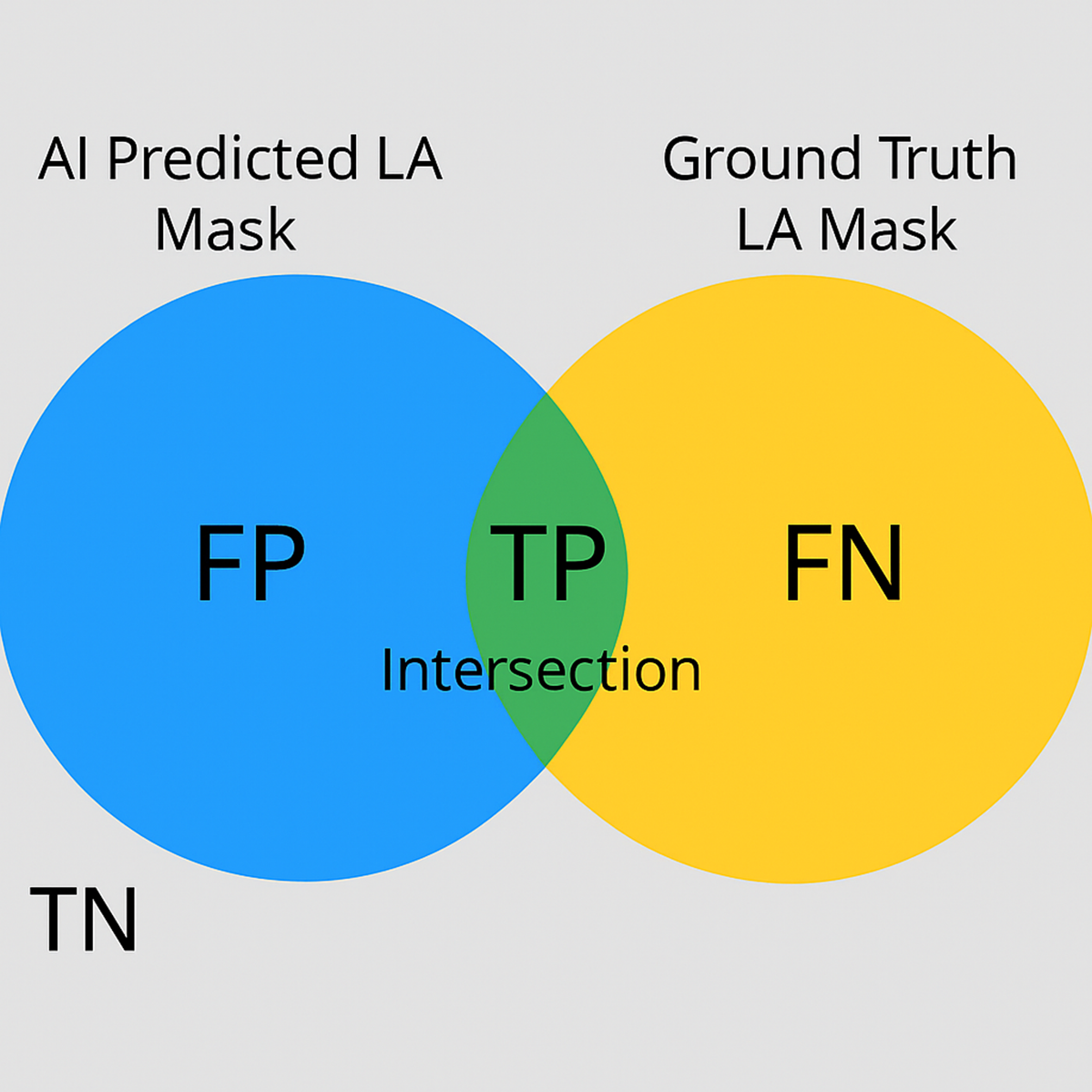
**

Figure S2. Voxel-wise Classification of Predicted and Ground Truth LA Masks. The Venn diagram illustrates the voxel-wise comparison between the AI-predicted leukoaraiosis (LA) mask (blue) and the ground truth LA mask (yellow).


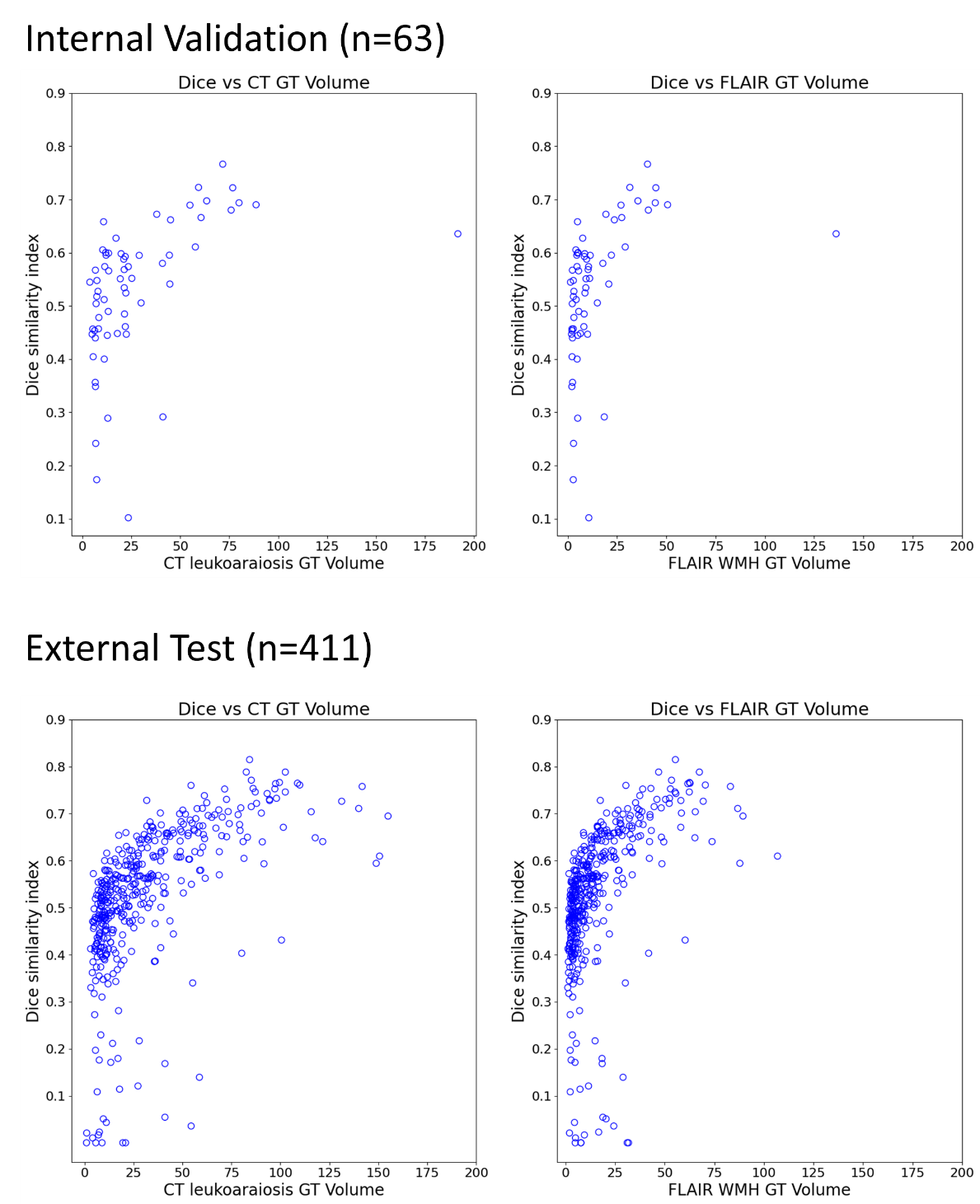


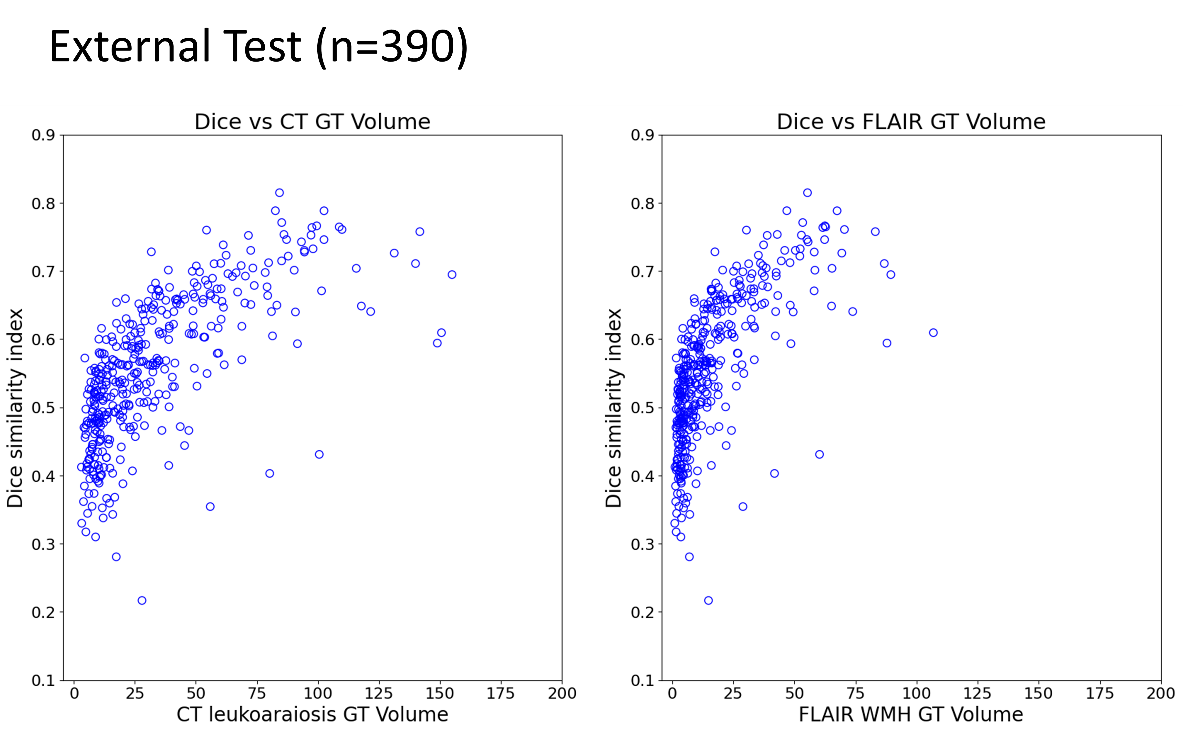


Figure S3. Distribution of Dice similarity coefficient according to leukoaraiosis volume on CT and white matter hyperintensity volume on FLAIR image.


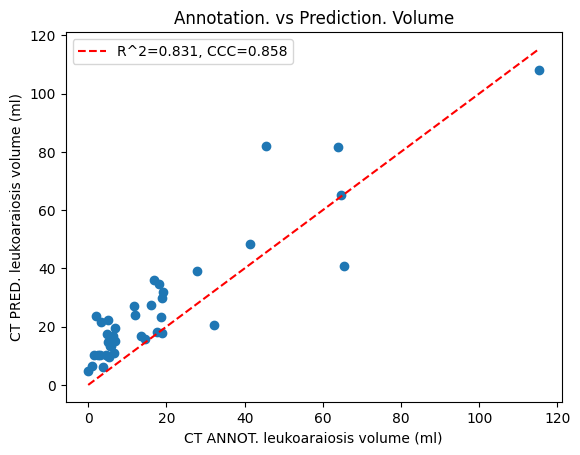
 Figure S4. Volumetric correlation between automatically segmented leukoaraiosis volume on CT and ground truth on CT by manual annotation in 40 randomly selected subjects in the external test dataset.


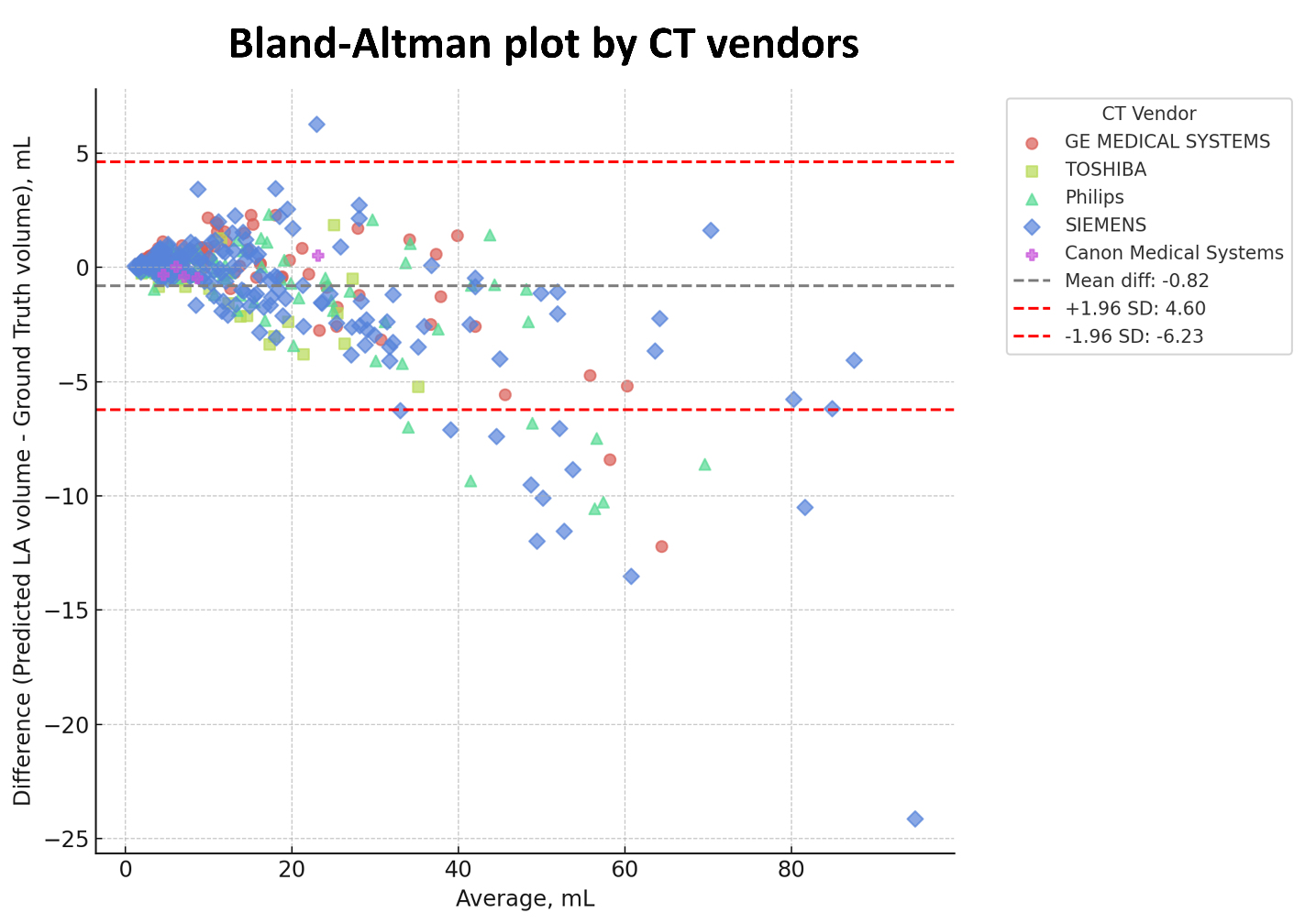


Figure S5. Bland–Altman plot comparing predicted leukoaraiosis (LA) volumes with ground truth volumes across CT vendors. The x-axis represents the average of the predicted and ground truth volumes, and the y-axis shows the difference (Predicted – Ground Truth), in milliliters. Each point corresponds to a case and is color-coded by CT vendor. The mean difference is −0.82 mL (black dashed line), with limits of agreement (±1.96 SD) at +4.60 mL and −6.23 mL (red dashed lines).


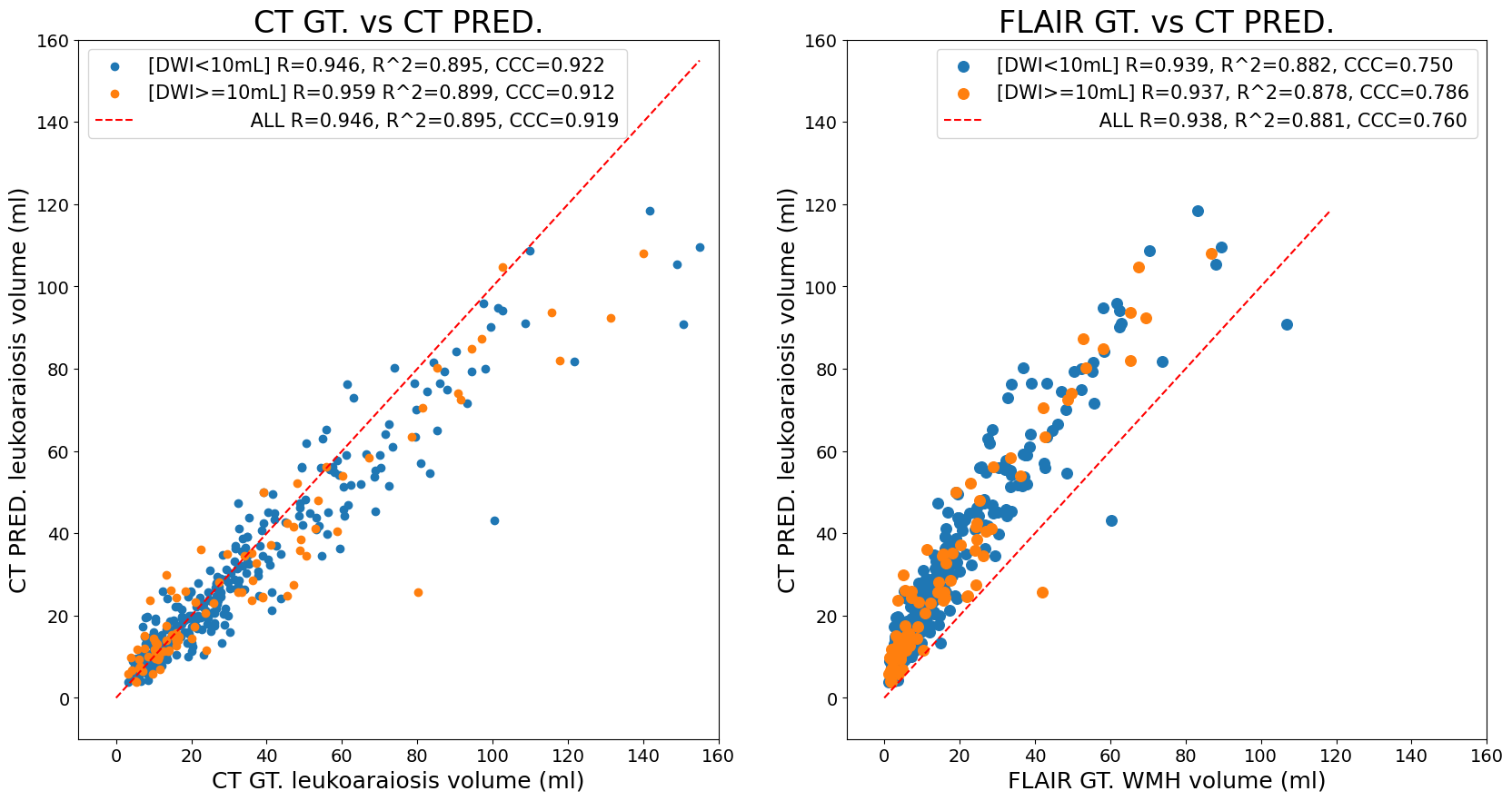


Figure S6. Volumetric correlation between automatically segmented leukoaraiosis volume on CT and ground truth on CT and MRI after stratification by infarct volume on DWI (<10 mL versus ≥10 mL) in the external test dataset.

Figure S7. Scatter plot showing the relationship between age and predicted leukoaraiosis volume on noncontrast CT. The black line represents the fit line, and the gray area indicates the 95% confidence interval. Linear regression was applied to subjects older than 40 years.
